# Constitutive immune function is not associated with fuel stores in spring migrating passerine birds

**DOI:** 10.1002/ece3.11516

**Published:** 2024-06-25

**Authors:** Shivani Ronanki, Arne Hegemann, Cas Eikenaar

**Affiliations:** ^1^ Department of Biology Lund University Lund Sweden; ^2^ Division of Toxicology Wageningen University and Research Wageningen The Netherlands; ^3^ Institute of Avian Research ‘Vogelwarte Helgoland’ Wilhelmshaven Germany

**Keywords:** fuel stores, immune function, movement ecology, optimal migration theory, spring migration, stopover ecology

## Abstract

Migratory birds may either upregulate their immune system during migration as they might encounter novel pathogens or downregulate their immune system as a consequence of trade‐offs with the resource costs of migration. Support for the latter comes not least from a study that reports a positive correlation in autumn migrating birds between fuel stores and parameters of innate and acquired immune function, that is, energy‐exhausted migrants appear to have lowered immune function. However, to our knowledge, no study has tested whether this pattern exists in spring migrating birds, which may face other trade‐offs than autumn migrants. Here, we investigate if in spring there is a relationship between fuel stores and microbial‐killing ability, a measure of innate immune function, and total immunoglobulin (IgY), a measure of acquired immune function, in four migrating bird species: chaffinches (*Fringilla coelebs*), dunnocks (*Prunella modularis*), song thrushes (*Turdus philomelos*) and northern wheatears (*Oenanthe oenanthe*). Our findings indicate no significant correlation between fuel stores and either microbial killing ability or IgY levels when considering all species collectively. When analysing species separately, we found a significant negative correlation between fuel stores and microbial‐killing ability in chaffinches and a positive correlation between fuel stores and IgY levels in wheatears. In song thrushes, but not in any of the other species, there was a significant negative correlation between relative arrival date and microbial‐killing ability and between arrival date and IgY levels. Sex did not affect immune function in any of the species. Our study suggests that the relationship between immune function and fuel stores may be different during spring migration compared to autumn migration. Differences in the speed of migration or pathogen pressure may result in different outcomes of the resource trade‐off between investment in immune function and migration among the seasons.

## Graphical abstract

Constitutive immune function is not linked to fuel stores in spring migrating birds. However, there is a negative relationship between immune function (% *Escherichia coli* killed) and arrival date. This suggests that there are seasonal variations in pathogen pressures and migratory speed which could impact the resource trade‐off between investment in immune function and demands of migration.

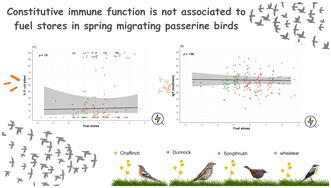

## 1 INTRODUCTION

Every year billions of birds migrate between their wintering and breeding grounds. During these migrations, birds encounter several challenges, which they have evolved to overcome (Maggini et al., 2021). Research shows that birds can regulate physiological processes such as immune function, metabolic rate and endocrine and neuro‐endocrine systems during migration (reviewed by Hegemann et al., 2019). Activation and maintenance of these processes along with long‐distance flight is costly and makes migration a highly resource‐demanding period. In order to keep up with these resource demands, birds are required to stop periodically to deposit fuel (Alerstam & Lindström, 1990). A recent review shows that physiological recovery and avoiding adverse weather conditions and spatiotemporal adjustments are also important functions of stopover besides refuelling (Schmaljohann et al., 2022).

Although stopovers are crucial for successful migration, they may facilitate the spread of diseases and increase the risk of encountering novel pathogens (Lickfett et al., 2018). Hence, a well‐functioning immune system during migration is important to minimize disease‐related mortality. However, it has been shown that endurance flight may negatively impact constitutive immune parameters in European Starlings, *Sturnus vulgaris* (Nebel et al., 2012), as well as in Red knots, *Calidris canutus* (Buehler et al., 2010), suggesting lower immuno‐competence in birds after a migratory flight. To (partly) compensate for this, migrants are able to boost immune function during stopover (Owen & Moore, 2008), within days of arrival at a stopover site (Eikenaar et al., 2023; Eikenaar, Hessler, & Hegemann, 2020).

If energy trade‐offs exist between migration and immune function, then a positive relationship between fuel stores and constitutive immune function during stopover is expected. Yet, relatively few studies have investigated if there is such a correlation between immune function and migrants' energy condition. A study by Eikenaar, Hegemann, et al. (2020) found that fuel stores are positively correlated with one innate and one acquired parameter of immune function in two sub‐species of wheatears, *Oenanthe oenanthe*, during autumn migration. A partly similar result was found during spring migration in four species of thrushes in a study by Owen and Moore (2008) where birds in poor energetic condition had lower leukocyte counts, a measure of acquired immunity, but found no differences in immunoglobulins, another measure of acquired immune function; they did not measure parameters of innate immune function. Hence, more research is needed to determine if this pattern is common among other bird species and if a positive relationship between innate immune function, the important first line of defence and fuel stores holds true for spring migration, which is much more hurried than autumn migration (Nilsson et al., 2013), mainly due to fewer stopovers in spring (Schmaljohann, 2018).

The existence of an (energy‐based) trade‐off between migration and immune function stems further from observations that such trade‐offs occur between immune function and/or immune responses and other energy‐demanding activities. This includes reproduction (e.g. Ardia et al., 2003; Deerenberg et al., 1997; Hasselquist & Nilsson, 2012; Hegemann et al., 2013; Ilmonen et al., 2000; Neggazi et al., 2016; Nordling et al., 1998; Norris & Evans, 2000), moult (e.g. Moreno‐Rueda, 2010) or periods of high thermoregulatory costs (e.g. Nord & Giroud, 2020) or food shortage (Buehler et al., 2009; Cornelius Ruhs et al., 2019; Demas & Nelson, 1998). Such trade‐offs can be directly related to available energy as both baseline (constitutive) immune function as well as immune responses require energy (Hasselquist & Nilsson, 2012; Klasing, 2007). Alternatively, and not mutually exclusive, the requirement for specific nutrients crucial for maintaining (constitutive) baseline immune function may make birds adjust their diet (e.g. Hegemann et al., 2013; Klasing, 2007) which could impact energy intake. Trade‐offs between immune function and other demanding activities like migration may also result in shifts within the immune system itself. During energy‐demanding times, redistributions within the immune system may occur, rather than an overall downregulation (e.g. Buehler et al., 2008; Lee, 2006). In either case, maintaining sufficient levels of baseline (constitutive) innate immune function could be especially important during migration if this allows an individual to avoid getting sick and thus having to face the energetic and behavioural costs (e.g. reduced activity and lethargy) accompanied by sickness behaviours (Burness et al., 2010; Lennon et al., 2023) which may result in severe fitness consequences.

In this study, we investigate if there is a relationship between fuel stores and one parameter of constitutive innate immune function, microbial‐killing ability, and one parameter of constitutive acquired immune function, the level of immunoglobulins (IgY), in four migrating bird species during spring stopover. These two parameters are relatively broad measures of innate and acquired immune function, respectively, and thus reflect an integrative measure of the immune system. We predict that there will be a positive correlation between fuel stores and immune parameters. As immune parameters usually differ between species and can differ between sexes, we also tested if the immune parameters differ between the four species included in this study and between the sexes. We furthermore tested if there is an effect of time within the migration season on the immune parameters as such an effect was documented in another study (Hegemann et al., 2022).

## 2 MATERIALS AND METHODS

### 2.1 Study site and study species

We studied four migrant bird species, chaffinches (*Fringilla coelebs*), dunnocks (*Prunella modularis*), song thrushes (*Turdus philomelos*) and northern wheatears (*Oenanthe oenanthe*, wheatear hereafter), on the Island of Helgoland (54°11′ N, 07°55′ E), 50 km off the German North Sea Coast. Chaffinches and dunnocks are diurnal migrants, whereas song thrushes and wheatears are nocturnal migrants. The birds were caught during daylight hours in the spring (March and April) of 2019. All birds sampled are assumed to be migrants, as only a few (or no) pairs of these species breed on Helgoland (Dierschke et al., 2011). A total of 50 song thrushes, 51 chaffinches, 45 wheatears and 51 dunnocks were captured for this study. Chaffinches were caught between 8 and 21 March, song thrushes between 7 and 31 March, wheatears between 26 March and 14 April and dunnocks between 4 and 20 March. We assume that the birds were captured soon after their arrival in Helgoland. We think this is a reasonable assumption because of the high continuous trapping effort (see below) as well as the fact that stopovers by passerines on Helgoland are typically in the range of days (e.g. Eikenaar et al., 2023).

### 2.2 Capture, sampling and measurements

All species except wheatears were caught using funnel traps. Wheatears were caught using mealworm‐baited spring traps. Blood was collected from the wing vein using Na‐heparinized microcapillaries. All birds were bled within 10 min of capture (range = 1–9.30 min, mean = 8.09 min), and hence before any expected impacts of handling stress on immune parameters (Zylberberg, 2015). The plasma was separated from the blood by centrifugation within 20 min of sampling for species caught in funnel traps and within 2 h of sampling for the wheatears. The plasma was stored at −20°C during the field season and at −50°C later on.

The birds were ringed and measured after blood sampling. Body mass was measured to nearest 0.1 g. Fat score was estimated on a scale of 0 (no fat) to 8 (furcular and abdomen bulging, and breast covered with fat) based on methods described by Kaiser (1993). Muscle score was estimated on a scale of 0 (sharp sternum and muscles depressed) to 3 (sternum difficult to distinguish due to rounded muscles) based on methods described by Bairlein (1994). Both measures are independent of body size and all measurements were consistently carried out by a single person. The sex of the birds was determined based on the plumage of wheatears and chaffinch, and molecular sexing was performed for song thrush and dunnock. All procedures were approved by the Ministry of Energy, Agriculture, the Environment, Nature and Digitalization, Schleswig‐Holstein, Germany (permit number V 242‐37068/2016).

### 2.3 Laboratory methods

All samples were randomized prior to the lab work and lab work was done blindly with respect to fuel store.

### 2.4 Microbial‐killing ability

To quantify constitutive innate immune function, the important less specific first line of defence (Janeway et al., 2005), we measured microbial‐killing ability. This assay measures the degree to which an individual's innate immune system can eradicate pathogens, in this case, the gram‐negative bacteria *Escherichia coli*. It thus represents a broad and integrative measure of innate immune function. We followed the protocol described by French and Neuman‐Lee (2012) along with some modifications by Eikenaar and Hegemann (2016). Volumes of 3 μL plasma and 4 μL of 1.06 × 10^6^ *E. coli* were finalized as optimal volumes for these species after running several test plates. The absorbance was measured using a FLUOstar Omega microplate reader. Microbial‐killing data that were slightly negative were set to 0 and values above 100% killing were set to 100, thus limiting the data to the range of 0–100.

### 2.5 Immunoglobulin (IgY) levels

To measure the constitutive part of acquired immune function, we measured immunoglobulin levels. We used the enzyme‐linked immunosorbent assay described by Sköld‐Chiriac et al. (2014) to determine the total level of immunoglobulins (antibodies) in plasma. For the standard curve, a plasma pool from adult jackdaws (*Corvus monedula*) was used. We used a seven‐step serial dilution (1000, 250, 62.5, 15.62, 3.90, 0.97 and 0.24) for the standard curve. Plasma samples of the focal species were diluted 1:600 and run in triplicate. A BioTek ELx50 plate washer was used to do the plate washes during the assay and the mean absorbance was quantified using a BioTek EL808 plate reader (measured in 10^3^ optical density per minute [mOD/min]). Antibody levels are calculated as the mean of the triplicates of each sample minus the mean value of the blanks and corrected for variation between plates according to the standard curves.

### 2.6 Data analysis

Due to limited plasma volumes, we could not run each immune assay for each individual. Final sample sizes were as follows: immunoglobulin levels: song thrush (*n* = 47), chaffinch (*n* = 46), wheatear (*n* = 40) and dunnock (*n* = 47); and microbial‐killing ability: song thrush (*n* = 49), chaffinch (*n* = 41), wheatear (*n* = 34) and dunnock (*n* = 39).

A measure we called ‘fuel stores’ was extracted from the variables fat score and muscle score for each species using principle component analysis. Both fat and muscle scores were included because migrating passerines depend on both fat (90%–95%) and protein (5%–10%) for energy during endurance flight (Jenni & Jenni‐Eiermann, 1998). The output for the PCA in form of the loadings and biplot is presented in Figure S1 and Table S1. Additionally, a body mass index was calculated by the formula mass divided by wing length. Since migration times differ between species, we standardized arrival dates of each species. We calculated a relative arrival date by giving 1 to the day the first individual of a given species was caught and counted upwards.

To test if there is an effect of fuel stores on immune parameters (IgY and microbial‐killing ability) across the four species, a generalized linear model approach was used. Species and sex were added as covariates given their documented influence on variation in immune function (Arriero et al., 2015; Tieleman et al., 2005). The body mass index was also added as a covariate as mass and size can influence immune cell proportions and metabolic rate (Cornelius Ruhs et al., 2021). Finally, relative arrival date was added as a covariate as early migrating individuals may differ in immune function from late migrating individuals (Hegemann et al., 2022).

Additionally, generalized linear models were also run to test the effect of fuel stores on immune parameters (IgY and microbial‐killing ability) in each species separately. For the model with the IgY data, a Gaussian error distribution was used. For the microbial‐killing data, a GLM with negative binomial distribution with a log link was used from the R package MASS. The body mass index, relative arrival date and sex were added as covariates to each model. Model predictions were met and model fit was assessed with the DHARma package. Model selection was performed by a stepwise elimination of non‐significant variables by using the drop.1 function in R.

## 3 RESULTS

**FIGURE 1:**
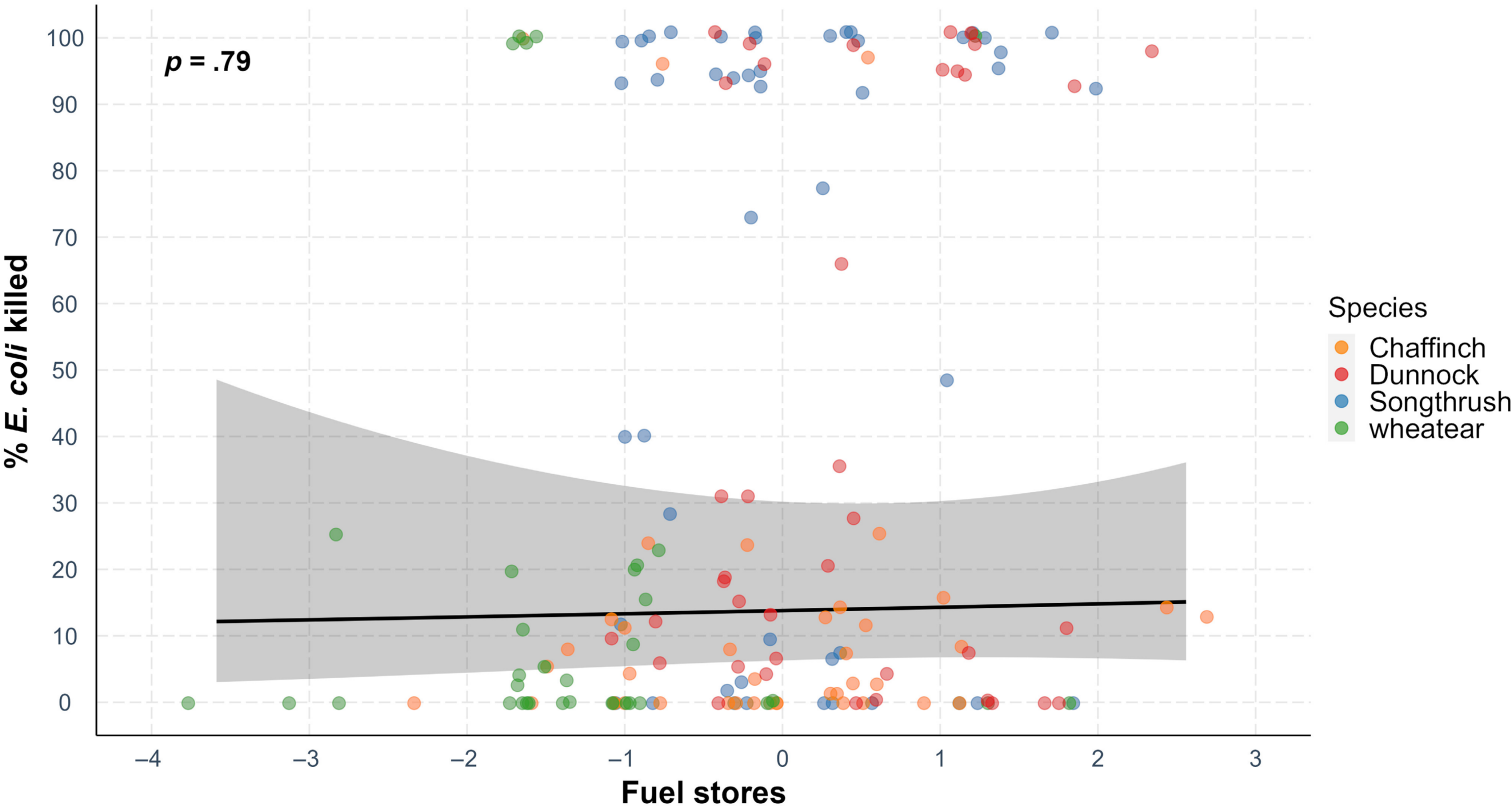
Microbial killing (% *E. coli* killed) ability and fuel stores in four species during spring migration. Points depict the raw data, while the line represents the prediction derived from the generalized linear model. Please note that the relationship between microbial killing ability and fuels stores was not significant (*p* = .79).

**FIGURE 2:**
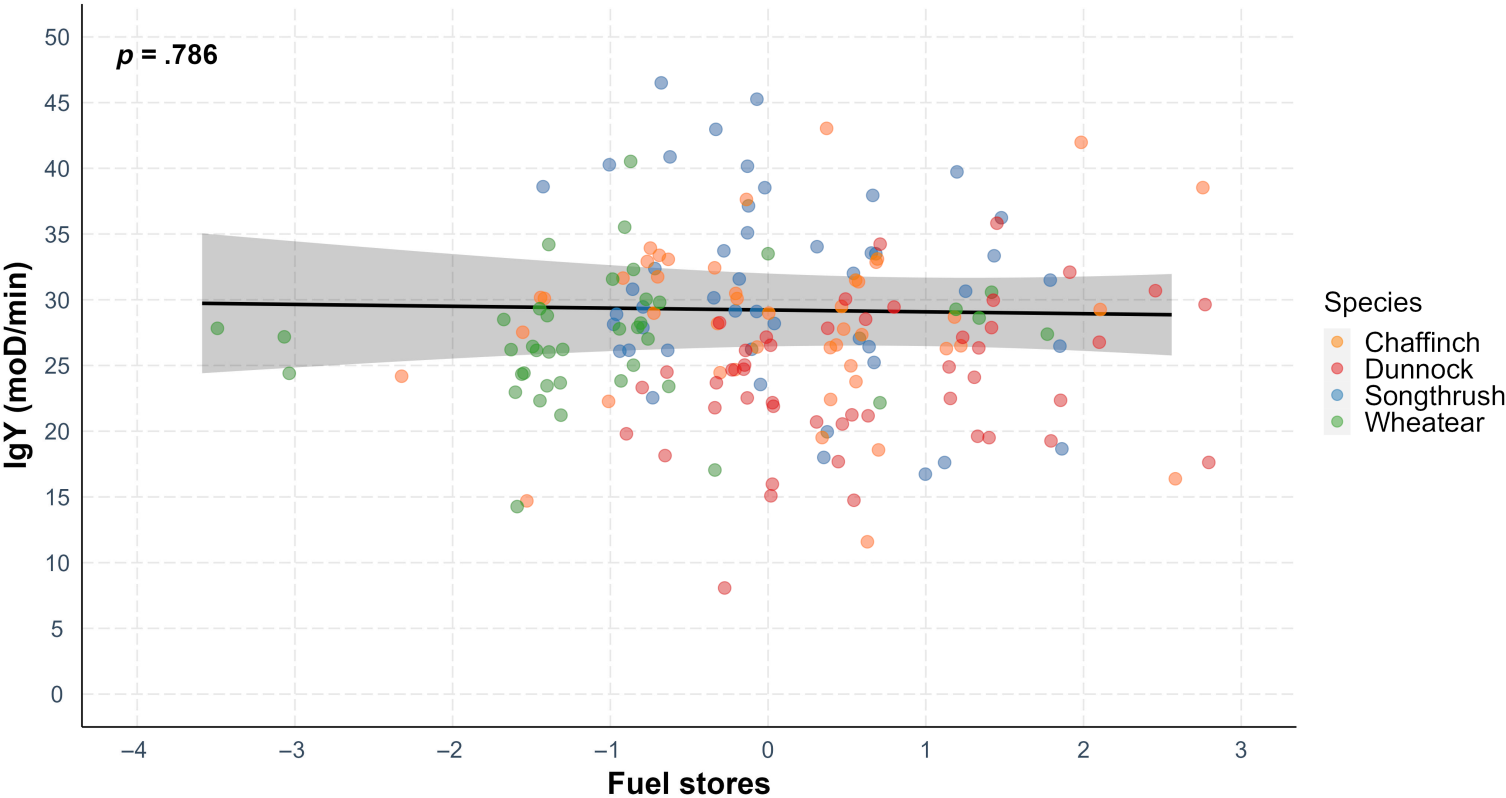
Immunoglobulin levels and fuel stores in four bird species during spring migration. Points depict the raw data, while the line represents the prediction derived from the generalized linear model. The relationship between IgY levels and fuels stores was not significant (*p* = .786).

There was no significant correlation between fuel stores and microbial‐killing ability (Table 1, Figure 1) or immunoglobulin (IgY) levels (Table 2, Figure 2) when all species were analysed in a single model. When species were analysed separately, there was a significant negative effect of fuel stores on microbial‐killing ability in chaffinches (Table S5) and a significant positive effect of fuel stores on IgY levels in wheatears (Table S10).

**TABLE 1:** Generalized linear model outputs for microbial‐killing ability (% *E. coli* Killed).

Microbial‐killing ability	Estimate	Standard error	*T*‐value	df	*p*‐Value
Intercept	2.466	1.083	2.276		
Fuel stores	0.0354	0.132	0.267	1	.799
Body size index	1.409	3.345	0.421	1	.745
Relative arrival day	−0.026	0.021	−1.212	1	.211
Sex	−0.020	0.278	−0.072	1	.943
Species				3	**.022**
Species: Dunnock	1.091	0.340	3.211		
Species: Song thrush	1.221	1.162	1.051		
Species: Wheatear	0.503	0.377	1.332		

*Note*: The table presents the effects of fuel stores, sex, species, relative arrival day and body mass index on microbial‐killing ability in four migrating passerine bird species during spring migration on Helgoland in the German Bay. Significant effects are indicated in bold. Statistical parameters are displayed from steps prior to dropping non‐significant variables from a model. The reference categories for sex and species are male and chaffinch, respectively.

**TABLE 2:** Generalized linear model outputs for IgY levels (moD/min).

IgY levels	Estimate	Standard error	*T*‐value	df	*p*‐Value
Intercept	24.227	4.035	6.00		
Fuel stores	−0.140	0.520	−0.271	1	.786
Body size index	17.970	13.600	1.321	1	.186
Relative arrival day	−0.113	0.088	−1.289	1	.197
Sex	1.140	1.070	1.065	1	.286
Species			−3.643	3	**.002**
Species: Dunnock	−4.657	1.278	−0.488		
Species: Song thrush	−2.296	4.703	−0.490		
Species: Wheatear	−0.698	1.426			

*Note*: The table presents the effects of fuel stores, sex, species, relative arrival day and body mass index on IgY levels in four migrating passerine bird species during spring migration on Helgoland in the German Bay. Significant effects are indicated in bold. Statistical parameters are displayed from steps prior to dropping non‐significant variables from a model. The reference categories for sex and species are male and chaffinch, respectively.

**FIGURE 3:**
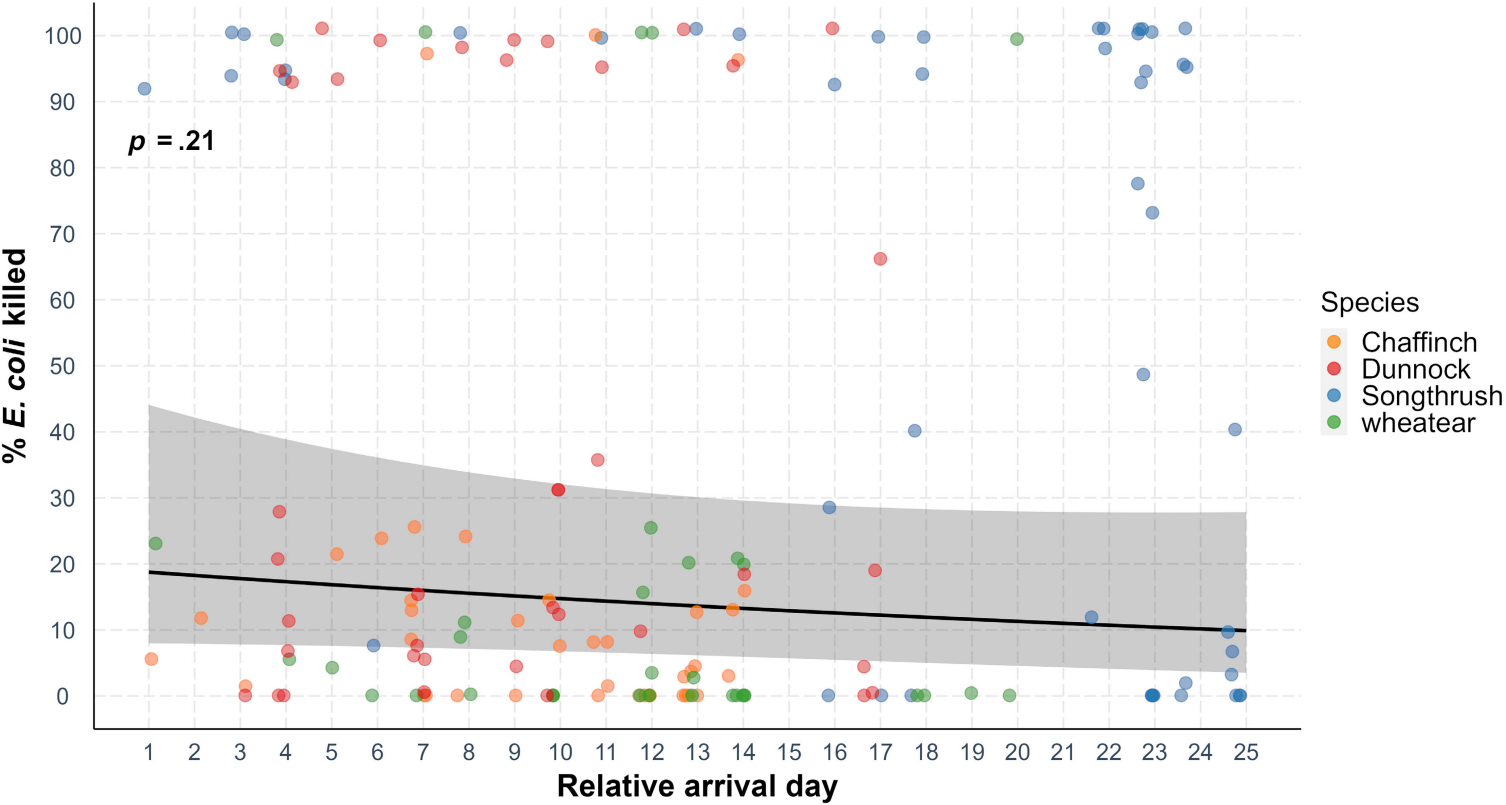
Microbial killing (% *E. coli* killed) ability and relative arrival date in four bird species during spring migration. Points depict the raw data, while the line represents the prediction derived from the generalized linear model. The relationship between microbial killing ability and relative arrival date was not significant (*p* = .21).

**FIGURE 4:**
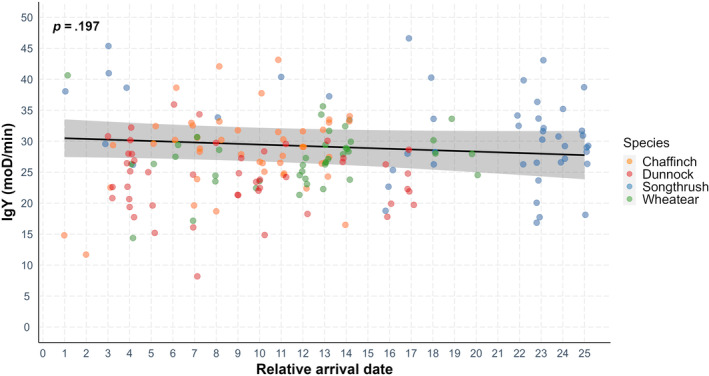
Immunoglobulin levels and relative arrival date in four bird species during spring migration points depict the raw data, while the line represents the prediction derived from the generalized linear model. The relationship between IgY levels and relative arrival date was not significant (*p* = .197).

When pooling all species, there was no correlation between microbial‐killing ability and relative arrival date (Table 1, Figure 3) or IgY levels and relative arrival date (Table 2, Figure 4). However, arrival date was negatively correlated with microbial‐killing ability as well as IgY levels in song thrushes (Tables S4 and S8).

**FIGURE 5:**
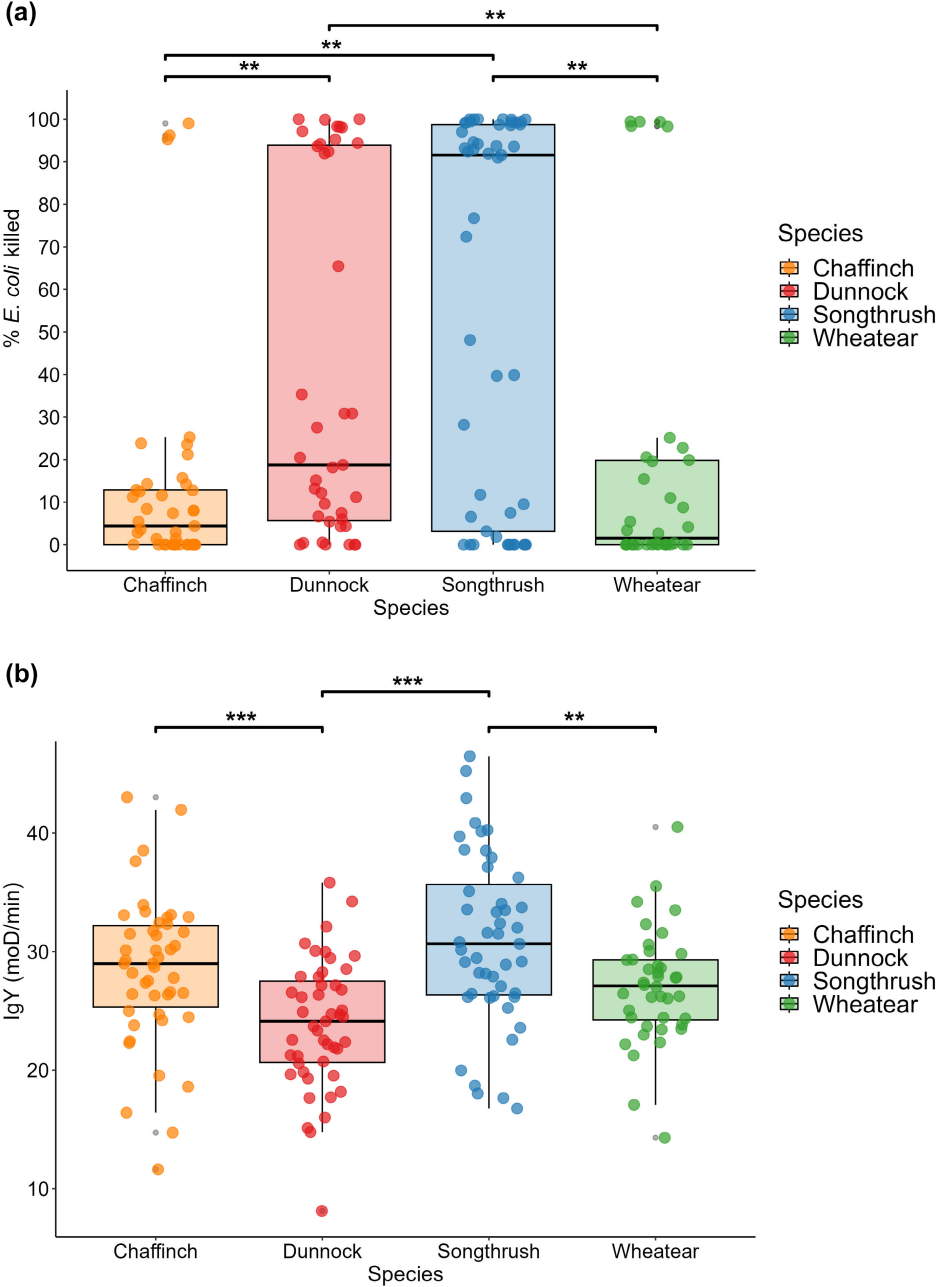
Box plots showing differences in (a) microbial killing ability (% *E. coli* killed) and (b) IgY levels (mOD/min) between four bird species measured at stopover on Helgoland, Germany. Open dots in the bar plots show the raw data, and lines indicate the lower quartile, median and upper quartile within each species.

Both microbial‐killing ability and IgY levels significantly differed between the species (Figure 5). Microbial‐killing ability was significantly different between the four species (Figure 5, χ^2^ = 20.43, df = 3, *p* < .001). A pairwise Wilcoxon test showed significantly higher microbial‐killing ability in dunnocks when compared to chaffinches (*p* = .004) and wheatears (*p* = .009). Song thrushes showed a significantly higher microbial‐killing ability than chaffinches (*p* = .004) and wheatears (*p* = .009). There was no significant difference in microbial‐killing ability between wheatears and chaffinches (*p* = .59), and dunnocks and song thrushes (*p* = .59). Similarly, immunoglobulin levels were significantly different between the four species (*f* = 11.25, df = 3, *p* < .001, Figure 5). A Tukey test showed that the IgY levels in dunnocks were significantly lower than IgY levels in chaffinches (*p* = .001) and song thrushes (*p* < .001). The IgY levels in wheatears were significantly lower than that in song thrushes (*p* = .01). There was no difference in levels of IgY between song thrushes and chaffinches (*p* = .19), wheatears and chaffinches (*p* = .68) or wheatears and dunnocks (*p* = .07). Sex did not influence microbial‐killing ability or IgY levels in any of the species.

Moreover, when the analysis was performed individually for muscle score and fat score, the results remained consistent, with the exception of an observed effect of muscle score on microbial‐killing activity in song thrushes (see Table S8).

## 4 DISCUSSION

We found that fuel stores were not correlated with an integrative measure of constitutive innate immune function (microbial‐killing ability) or with a measure of constitutive acquired immune function (immunoglobulin levels) when species were analysed collectively. Only when analysing species separately, we found a negative correlation between bacteria‐killing capacity and fuel stores in chaffinches and a negative correlation between immunoglobulin levels and fuel stores in wheatears.

This finding is contrary to what was found in wheatears during autumn migration where there was a positive correlation between fuel stores and both these two same parameters of immune function (Eikenaar, Hegemann, et al., 2020). Interestingly and similar to this current study, there was no correlation between energetic condition and immunoglobulin levels in spring migrating thrushes in the Americas either (Owen & Moore, 2008). However, Owen and Moore (2008) did observe a negative relationship between energetic condition and total white blood cell count as well as one of five leucocyte types. They did not measure innate immune function. Taken together those studies may suggest that while there is a correlation between immune function and fuel stores during autumn migration, such a relationship may at most be weak in spring migrating birds.

A potential explanation for the differences between autumn and spring migrating birds could lie in seasonal differences in migration speed that impact resource allocation between immune function and the demands of migration. There is evidence that progress during spring migration is much faster than during autumn migration as birds travel with fewer stopovers in spring (Nilsson et al., 2013; Schmaljohann, 2018). The reason for a more hurried spring migration is that there is a fitness advantage for arriving early at the breeding grounds; individuals that arrive earlier at the breeding grounds are more successful in finding a territory and a mate (Aebischer et al., 1996; Kokko, 1999). Perhaps, spring migrants tend to invest their resources (energy and nutrients for fuel) more into timely arrival at the breeding site rather than in immune function compared to autumn migrants. Fewer stopovers may also mean that spring migrants have less opportunity to recover their immune function during stopover after strenuous endurance flights (Eikenaar et al., 2023; Eikenaar, Hessler, & Hegemann, 2020). The duration of stopovers unlikely limits recovery of immune function, as we have previously shown that such recovery can occur within 2 days (Eikenaar, Hessler, & Hegemann, 2020). Some support for a seasonal difference in immune function comes from comparing values from the current and previous studies. Eikenaar, Hegemann, et al. (2020) found that in autumn migrating wheatears, IgY levels ranged from 45 to 60 mOD/min in autumn, whereas in the current spring study, these typically ranged from 20 to 35 mOD/min (Figure 5). Similarly, in comparison to an autumnal study by Hegemann et al. (2018), the mean IgY levels for dunnocks, song thrushes and chaffinches were much lower in the current spring study. It is important to note though that direct comparisons should be made with caution, as the assays in the various studies were performed with different standards and dilutions. Nonetheless, they are suggestive of a lower (investment in) immune function in spring than autumn, which could help to explain why in the current spring stopover study we did not observe relationships between migrants' fuel stores and parameters of immune function.

Three other, not mutually exclusive hypotheses might further help explain why there is no correlation between immune function and fuel loads in spring migrating birds and why they may have lower immune function than birds during autumn migration. All three relate to pathogen pressure, that is, the risk of encountering pathogens and becoming sick. First, densities of birds are usually much lower in spring than during autumn, especially when considering that our study location is relatively close to our target species' breeding locations because most annual mortality happens between autumn and spring (Klaassen et al., 2014; Leyrer et al., 2013; Sillett & Holmes, 2002). Lower densities of other birds will decrease contact rate and hence the risk of transmission of infectious diseases. Second, prevalence of diseases is often higher during autumn than during spring (Latorre‐Margalef et al., 2014; Van Dijk et al., 2014), which also reduces the risk of infection. Third, during autumn birds are on their way to southerly wintering grounds, which supposedly harbour more pathogens than the northerly breeding grounds to which birds are heading during spring (Connor et al., 2020). All three hypotheses reduce the risk of disease contact and hence the need for strong constitutive immune function (Hegemann et al., 2012; Horrocks et al., 2011, 2015). Hence, this may allow birds to invest more in migration and less in immune function during spring migration compared to autumn migration. Finally, we cannot rule out the possibility that the limited sample size for each species could potentially contribute to the lack of statistical power, which in turn resulted in non‐significant correlation between fuel stores and immune function even though we tried to maximize statistical power by pooling species.

We found no significant correlation between immune function and relative arrival date among all species pooled. However, there was a significant negative correlation between arrival date and microbial‐killing ability and arrival date IgY levels in song thrushes; individuals that arrived in Helgoland early in the migration season had higher microbial‐killing ability and IgY levels compared to the later arriving individuals. We can only speculate on the reason(s) for these relationships, but perhaps birds arriving at stopover earlier are of superior quality than the ones arriving later. There is well‐established association between early arrival and superior quality (Kokko, 1999). Arrival time is correlated with factors such as song length (Arvidsson & Neergaard, 1991; Lampe & Espmark, 1994; Nyström, 1997), brightness in colour (Slagsvold & Lifjeld, 1988) as well as haematocrit levels (Saino, Cuervo, Krivacek, et al. 1997; Saino, Cuervo, Ninni, et al. 1997). It is also possible that individuals wintering further south passed by Helgoland later, and had lower immune function because of their longer migratory flights. We furthermore observed significant differences in microbial‐killing ability as well as IgY levels among the species, which is in line with previous studies (e.g. Eikenaar et al., 2023; Hegemann et al., 2022; Tieleman et al., 2005). Variations in immune function between species can have multiple reasons including differences in risk of infection, phylogenetic history, migration strategy, etc. It is beyond the scope of our study to discuss or even disentangle possible reasons, but the data provided might prove valuable for future studies or meta‐analyses.

In conclusion, our study showed that unlike during autumn migration, during spring migration fuel stores in dunnocks, song thrushes, wheatears and chaffinches are not related to immune function. Moreover, the current and previous studies hint at differential seasonal investment in immune function, which could be further investigated in focused comparative studies.

## AUTHOR CONTRIBUTIONS

**Shivani Ronanki:** Formal analysis (equal); investigation (equal); visualization (equal); writing – original draft (equal). **Arne Hegemann:** Conceptualization (equal); funding acquisition (equal); investigation (equal); methodology (equal); supervision (equal); writing – original draft (equal); writing – review and editing (equal). **Cas Eikenaar:** Conceptualization (equal); data curation (equal); funding acquisition (equal); investigation (equal); methodology (equal); project administration (equal); supervision (equal); writing – original draft (equal); writing – review and editing (equal).

## CONFLICT OF INTEREST STATEMENT

We declare no conflict of interest.

## Supporting information


Data S1:

## Data Availability

The data that support the findings of this study are openly available in dryad (reviewer link) at https://datadryad.org/stash/share/4MX1lEtbx5dAtXFtnPZYhG2Wor7_sEZ17q4viPe_L1o.
