# Supplementary material for: Constitutive immune function is not associated with fuel stores in spring migrating passerine birds
Source: Ecol Evol. 2024 Jun 25;14(6):e11516. doi: 10.1002/ece3.11516 (PMC11199120; doi:10.1002/ece3.11516)
Supplement: Supplementary file 1 — Data S1: [file ECE3-14-e11516-s001.docx]

**Supplementary material to**

**Constitutive immune function is not associated with fuel stores in spring migrating passerine birds**

**Shivani Ronanki^1,2*^, Arne Hegemann^1*^, Cas Eikenaar^3^**

^1^Biology Department of Biology, Lund University, Ecology Building, Lund SE-223 62, Sweden

**^2^** Division of toxicology, Wageningen University and Research, Wageningen 6708 WE, The Netherlands

**^3^**Institute of Avian Research “Vogelwarte Helgoland”, 26386 Wilhelmshaven, Germany

*Corresponding author’s email-address: [shivani.ronanki@wur.nl](mailto:shivani.ronanki@wur.nl); [arne.hegemann@biol.lu.se](mailto:arne.hegemann@biol.lu.se)

Principal component analysis output

Table S1: Principal component analysis output overview of the analysis of the two variables fat score and muscle score in four species of passerine birds measuring during spring migration at a stopover site on Helgoland, Germany.

|  | Comp.1 | Comp.2 |
| --- | --- | --- |
| Standard deviation | 1.142 | 0.833 |
| Proportion of Variance | 0.652 | 0.347 |
| Cumulative proportion | 0.652 | 1.000 |
| SS loadings | 1.0 | 1.0 |
| Proportion Var | 0.5 | 0.5 |
| Cumulative Var | 0.5 | 1.0 |


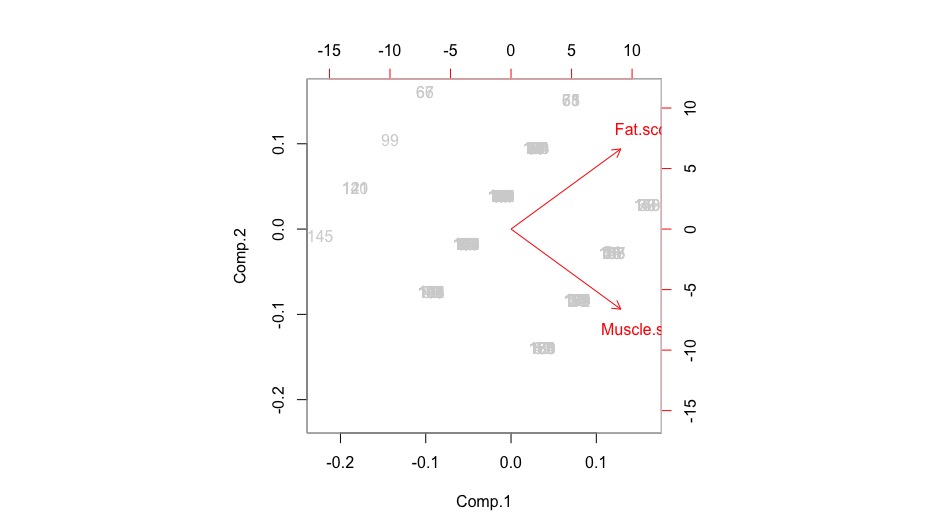


Figure S1: Biplot showing relationship between two variables fat score and muscle score in four species of passerine birds measuring during spring migration at a stopover site on Helgoland, Germany.

TableS2: .General linear model output for models testing the effects of muscle score, sex, and Julian day on BKA levels per species,and fat score, sex and Julian day on microbial killing ability per species. Significant effects are marked in bold. Statistical parameters displayed are from steps prior to dropping non-significant terms from the model. Birds were caught during spring migration on Helgoland/Germany

|  |  |  |  |  |  |  |  | |  | |  |
| --- | --- | --- | --- | --- | --- | --- | --- | --- | --- | --- | --- |
| Model/Species | Estimate | Standard Error | T-value | P-value | Model/Species | Estimate | | Standard Error | | T-value | P-value |
| **Chaffinch**  Muscle score  Sex  Julian day | -10.997  3.651  0.043 | 10.409  10.761  1.248 | -1.057  0.339  0.035 | 0.298  0.736  0.972 | **Chaffinch**  Fat score  Sex  Julian day | -0.502  6.780  -0.220 | | 4.126  11.284  1.253 | | -0.122  0.601  -0.175 | 0.904  0.552  0.862 |
| **Dunnock**  Muscle score  Sex  Julian day | 23.269  -14.971  0.736 | 14.112  18.085  1.586 | 1.649  -0.828  0.446 | 0.108  0.413  0.645 | **Dunnock**  Fat score  Sex  Julian day | 7.830  -11.759  -0.474 | | 10.502  18.581  1.647 | | 0.746  -0.633  0.288 | 0.461  0.531  0.775 |
| **Song thrush**  **Muscle score**  Sex  **Julian day** | 32.987  -6.920  -2.695 | 15.045  12.533  0.860 | 2.192  -0.552  -3.136 | **0.033**  0.583  **0.003** | **Song thrush**  Fat score  Sex  **Julian day** | -3.493  -9.651  -2.174 | | 8.128  13.116  0.887 | | -0.430  -0.736  -2.450 | 0.669  0.465  **0.018** |
| **Wheatear**  Muscle score  Sex  Julian day | 12.296  2.881  -0.6204 | 14.847  16.854  1.485 | 0.828  0.171  -0.418 | 0.414  0.865  0.679 | **Wheatear**  Fat score  Sex  Julian day | -4.356  3.815  -1.171 | | 7.654  16.981  1.432 | | -0.569  0.225  -0.818 | 0.574  0.824  0.420 |

Table S3: Generalised linear model output for models testing the effects of muscle score, sex, and Julian day on IgY levels per species,and fat score, sex and Julian day on IgY per species. Significant effects are marked in bold. Statistical parameters displayed are from steps prior to dropping non-significant terms from the model.

|  |  |  |  |  |  |  |  | |  | |  |
| --- | --- | --- | --- | --- | --- | --- | --- | --- | --- | --- | --- |
| Model/Species | Estimate | Standard Error | T-value | P-value | Model/Species | Estimate | | Standard Error | | T-value | P-value |
| **Chaffinch**  Muscle score  Sex  Julian day | 2.940  2.428  0.458 | 2.002  2.502  0.279 | 1.468  0.970  1.638 | 0.149  0.337  0.109 | **Chaffinch**  Fat score  Sex  Julian day | -0.697  2.473  0.429 | | 0.980  2.653  0.292 | | -0.712  0.932  1.471 | 0.480  0.357  0.149 |
| **Dunnock**  Muscle score  Sex  Julian day | 3.174  2.026  0.002 | 1.613  1.961  0.174 | 1.968  1.034  0.015 | 0.05  0.307  0.988 | **Dunnock**  Fat score  Sex  Julian day | 0.310  2.283  -0.092 | | 1.026  2.047  0.178 | | 0.303  1.115  -0.515 | 0.763  0.271  0.609 |
| **Song thrush**  Muscle score  Sex  **Julian day** | -1.114  -2.547  -0.320 | 2.574  2.058  0.140 | -0.433  -1.238  -2.276 | 0.667  0.222  **0.027** | **Song thrush**  Fat score  Sex  **Julian day** | -1.873  -2.588  -0.296 | | 1.154  2.001  0.136 | | -1.623  -1.293  -2.175 | 0.111  0.202  **0.035** |
| **Wheatear**  Muscle score  Sex  Julian day | 1.224  -2.682  0.174 | 1.749  1.726  0.178 | 0.700  -1.554  0.978 | 0.489  0.129  0.334 | **Wheatear**  Fat score  Sex  Julian day | 1.512  -2.700  0.182 | | 0.887  1.669  0.168 | | 1.703  -1.618  1.087 | 0.097  0.114  0.284 |

| **Song Thrush**  **Microbial killing ability** | **Estimate** | | **Standard Error** | **T-value** | **df** | **P-value** |
| --- | --- | --- | --- | --- | --- | --- |
| Intercept  Fuel stores  Body mass index  **Arrival day**  Sex | | 2.563  0.029  -0.307  -0.005  0.0158 | 0.163  0.0170.2470.001  0.027 | 15.651  1.741  -1.244  -3.082  0.571 | 1  1  1  1 | 0.0819  0.567  0.210  **0.002** |

Table S4: Generalised linear model output for testing the effects of fuel stores, body mass index, Arrival day and sex on microbial killing ability in Song thrushes. The reference catergory for sex is male. Arrival day was found to have a significant negative effect on the microbial killing capacity in song thrushes

*Table S5: Generalised linear model output for testing the effects of fuel stores, body size index, Arrival day and sex on microbial killing ability in Chaffinches. The reference category for sex is male. Fuel stores and body mass index had a significant effect on microbial killing ability in Chaffinches.*

| **Chaffinch**  **Microbial killing ability** | **Estimate** | **Standard Error** | **T-value** | **df** | **P-value** |
| --- | --- | --- | --- | --- | --- |
| Intercept  **Fuel stores**  **Body mass index**  Arrival day  Sex | 1.799  -0.0461  1.912  0.002  -0.041 | 0.224  0.0201  0.766  0.004  0.039 | 8.00  -2.293  2.496  0.495  -1.076 | 1  1  1  1 | **0.021**  **0.012**  0.620  0.280 |

*Table S6: Generalised linear model output for testing the effects of fuel stores, body mass index, Arrival day and sex on microbial killing ability in Wheatear. The reference catergory for sex is male. None of the covariates influenced the microbial killing ability in Wheatears.*

| **Wheatear**  **Microbial killing ability** | **Estimate** | **Standard Error** | **T-value** | **df** | **P-value** |
| --- | --- | --- | --- | --- | --- |
| Intercept  Fuel stores  Body mass index  Arrival day  Sex | 7.523  -0.135  10.940  -0.027  -0.017 | 3.256  0.260  12.420  0.0425  0.477 | 2.31  -0.521  0.881  -0.639  -0.036 | 1  1  1  1 | 0.602  0.377  0.5140.971 |

*Table S7: Generalised linear model output for testing the effects of fuel stores, body mass index, Arrival day and sex on microbial killing ability in Dunnock. The reference category for sex is male.* *None of the covariates influenced the microbial killing ability in Dunnocks.*

| **Dunnock**  **Microbial killing ability** | **Estimate** | **Standard Error** | **T-value** | **df** | **P-value** |
| --- | --- | --- | --- | --- | --- |
| Intercept  Fuel stores  Body mass index  Arrival day  Sex | 10.215  0.447  -3.84  0.023  0.376 | 2.529  0.249  7.994  0.039  0.446 | 4.039  1.792  0.481  0.597  0.841 | 1  1  1  1 | 0.072  0.624  0.551  0.394 |


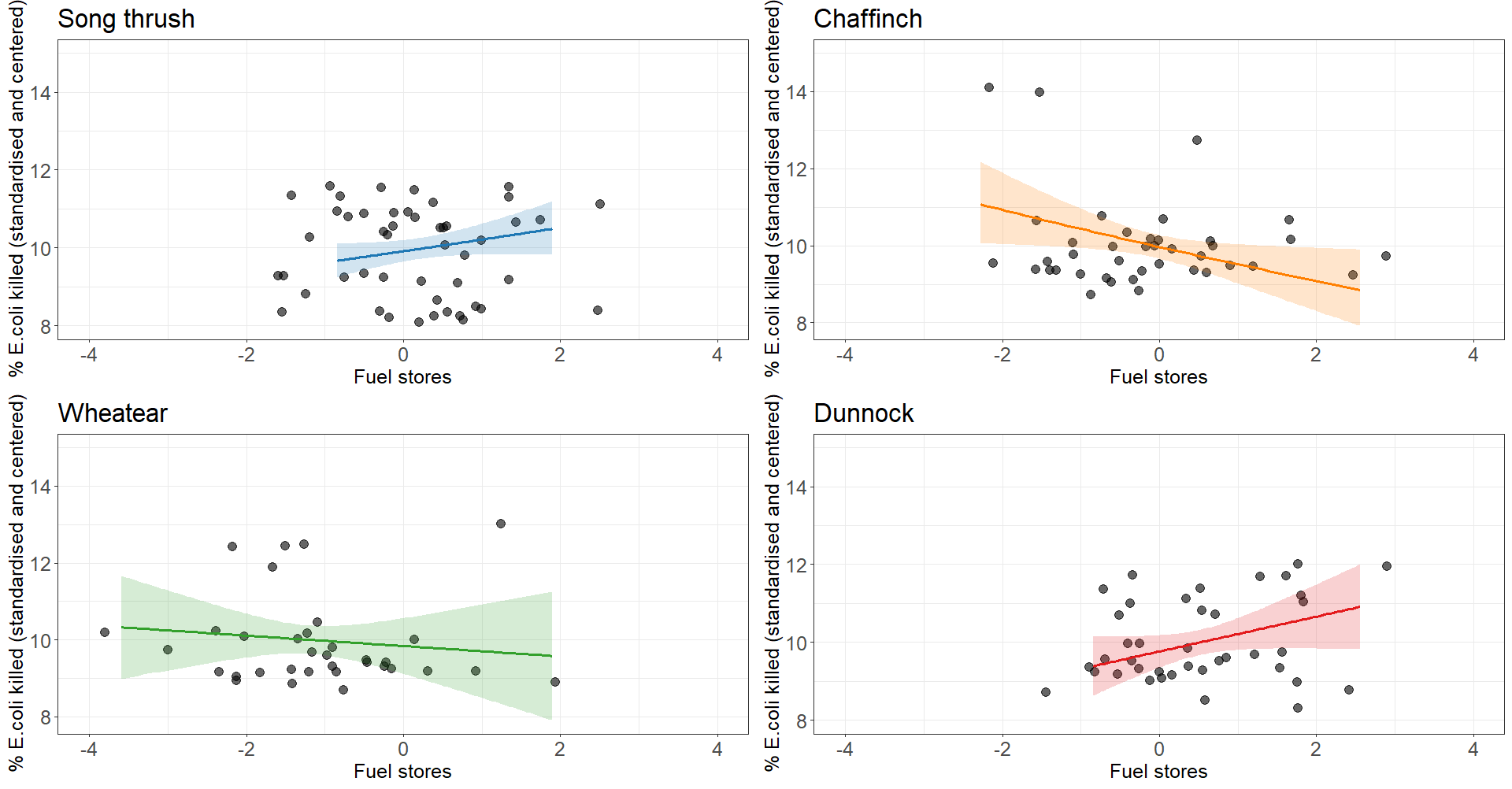


Figure S2: : Correlation between Microbial Killing ability and fuel stores in Four Species during spring migration. Description: This scatter plot illustrates the interaction between microbial killing (% E. coli killed) and the relative arrival date across four distinct species. The line represents the prediction from the generalized linear species specific model and the dots represent the raw data points.


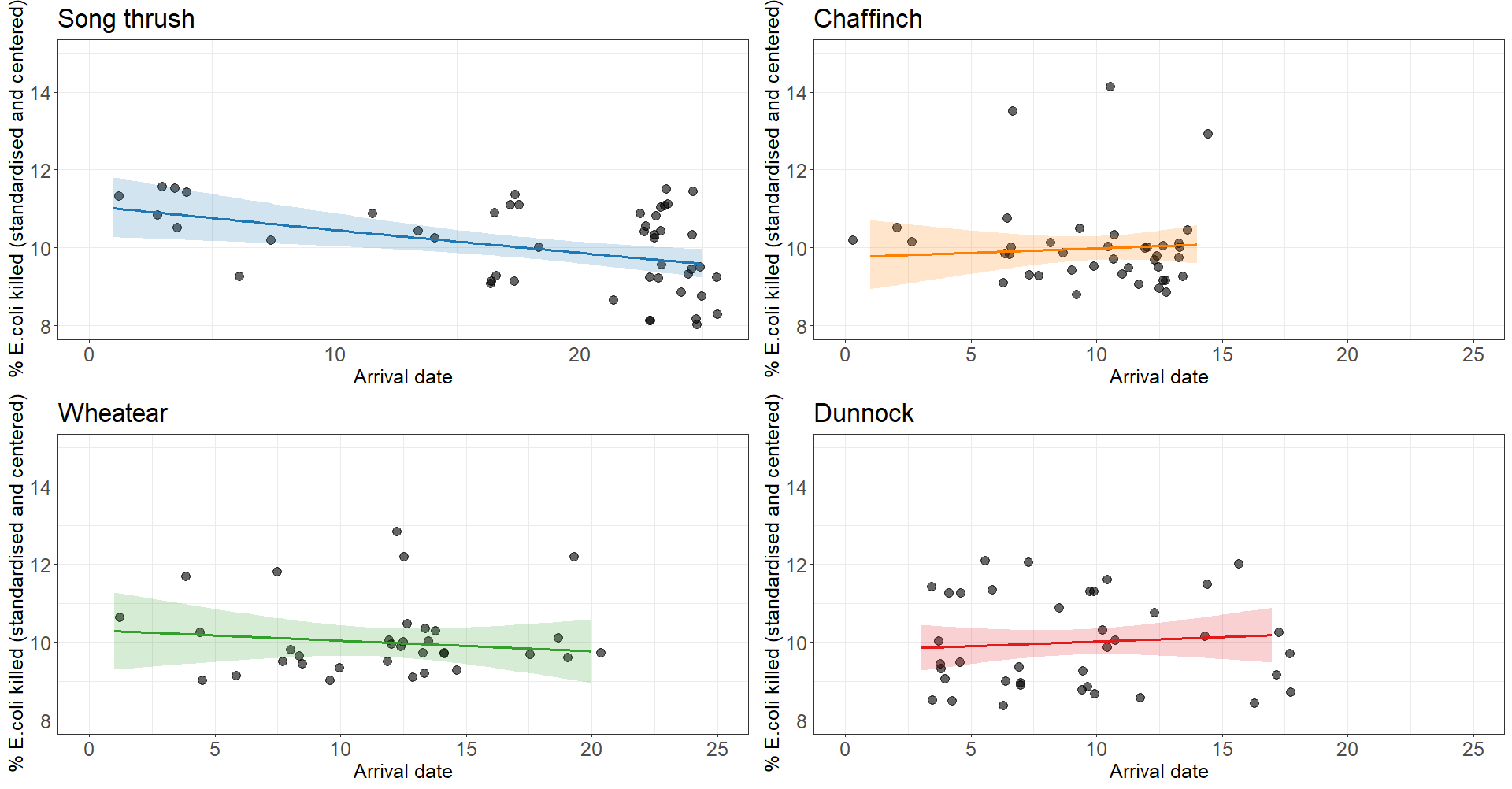


*Figure S3: : Correlation between Microbial Killing ability and relative arrival date in Four Species during spring migration. Description: Each scatter plot illustrates the interaction between microbial killing (% E. coli killed) and the arrival date across four distinct species. The line represents the prediction from the generalized linear species specific model, and the dots represent the raw data points.*

*Table S8: Generalised linear model output for testing the effects of fuel stores, body mass index, Arrival day and sex on IgY levels in Song thrushes. The reference catergory for sex is male. Arrival day was found to have a significant negative effect on IgY levels in song thrushes*

| **Song Thrush**  **IgY levels** | **Estimate** | **Standard Error** | **T-value** | **df** | **P-value** |
| --- | --- | --- | --- | --- | --- |
| Intercept  Fuel stores  Body mass index  **Arrival day**  Sex | *16.938*  *-2.177*  *30.478*  *-0.276*  *2.866* | *10.730*  *1.198*  *16.983*  *0.137*  *2.013* | *1.579*  *-1.816*  *1.795*  *-2.014*  *1.424* | *1*  *1*  *1*  *1* | *0.069*  *0.072*  ***0.044***  *0.154* |
|  |  |  |  |  |  |

*Table S9: Generalised linear model output for testing the effects of fuel stores, body mass index, Arrival day and sex on IgY levels in Chaffinches. The reference catergory for sex is male.* *None of the covariates influenced the microbial killing ability in Dunnocks.*

| **Chaffinch**  **IgY levels** | **Estimate** | **Standard Error** | **T-value** | **df** | **P-value** |
| --- | --- | --- | --- | --- | --- |
| Intercept  Fuel stores  Body mass index  Arrival day  Sex | *25.328*  *0.597*  *-6.251*  *0.535*  *-1.672* | *13.369*  *1.180*  *45.428*  *0.300*  *2.586* | *1.895*  *0.506*  *-0.138*  *1.778*  *-0.647* | *1*  *1*  *1*  *1* | *0.612*  *0.517*  *0.075*  *0.890* |

| **Wheatear**  **IgY levels** | **Estimate** | **Standard Error** | **T-value** | **df** | **P-value** |
| --- | --- | --- | --- | --- | --- |
| Intercept  **Fuel stores**  Body mass index  Arrival day  Sex | *52.758*  *2.754*  *-108.62*  *0.248*  *2.015* | *15.281*  *1.219*  *59.194*  *0.171*  *1.690* | *3.452*  *2.258*  *-1.835*  *1.451*  *1.192* | *1*  *1*  *1*  *1* | ***0.023***  *0.066*  *0.146*  *0.233* |

*Table S10: Generalised linear model output for testing the effects of fuel stores, body mass index, Arrival day and sex on IgY levels in Wheatears. The reference catergory for sex is male. Fuel stores had a significant positive correlation on IgY levels in Wheatears.*

*Table S11: Generalised linear model output for testing the effects of fuel stores, body mass index, Arrival day and sex on IgY levels in Dunnocks. The reference catergory for sex is male. None of the covariates influenced the microbial killing ability in Dunnocks.*

| **Dunnock**  **IgY levels** | **Estimate** | **StandardError** | **Tvalue** | **df** | **P-value** |
| --- | --- | --- | --- | --- | --- |
| Intercept  Fuel stores  Bodymassindex  Arrival day  Sex | *2.823*  *1.922*  *-1.75*  *2.507*  *-2.167* | *1.191*  *1.200*  *4.053*  *1.880*  *2.085* | *2.371*  *1.601*  *-0.432*  *0.00*  *-1.03* | *1*  *1*  *1*  *1* | *0.109*  *0.665*  *0.999*  *0.298* |


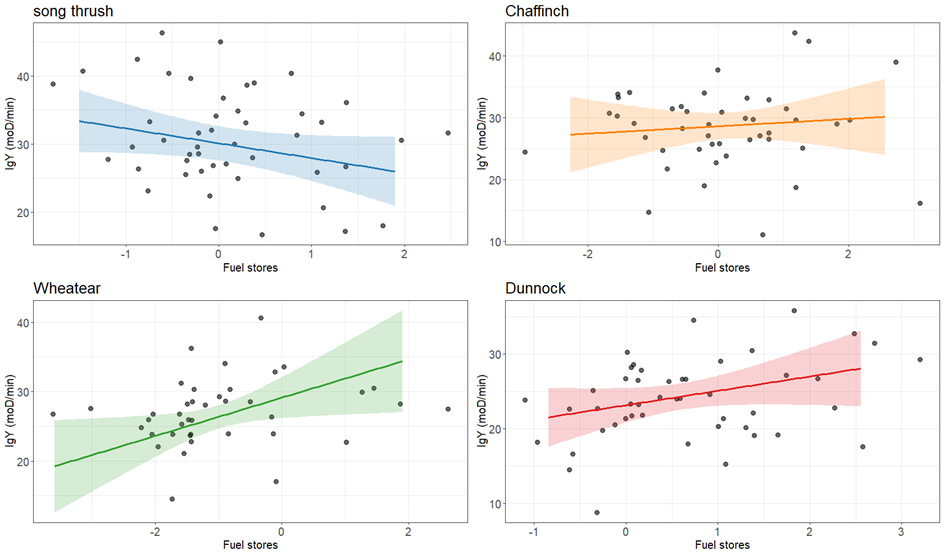


Figure S4: : Correlation between IgY levels and fuel stores in Four Species during spring migration. Description: This scatter plot illustrates the interaction between IgY and the relative arrival date across four distinct species. The line represents the prediction from the generalized linear species specific model and the dots represent the raw data points.


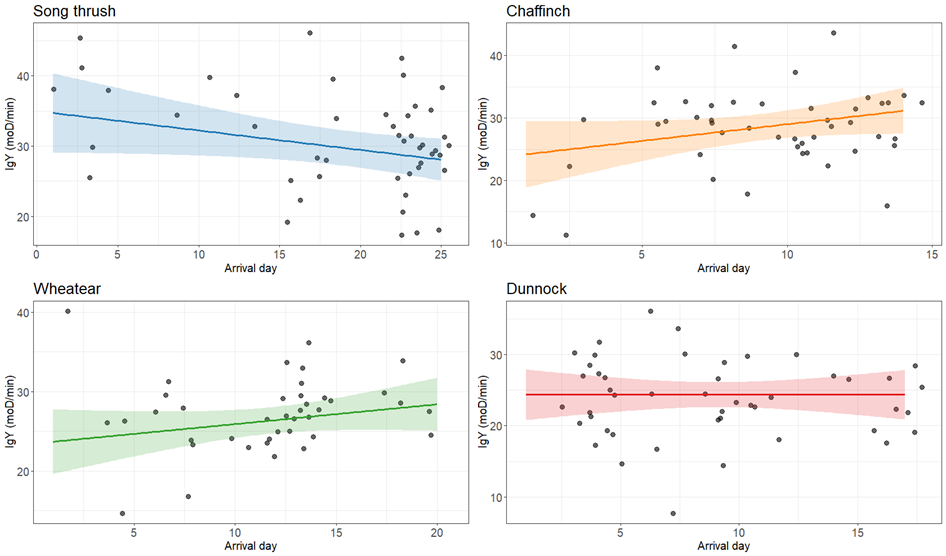


*Figure S5: : Correlation between IgY levels and relative arrival date in Four Species during spring migration. Description: Each scatter plot illustrates the interaction between IgY and the arrival date across four distinct species. The line represents the prediction from the generalized linear species specific model, and the dots represent the raw data points.*
